# Effective palliative radiofrequency ablation for tumors causing pain, numbness and motor function disorders: case series

**DOI:** 10.1186/1756-0500-7-765

**Published:** 2014-10-28

**Authors:** Shinichi Hamamoto, Toshiyuki Matsuoka, Tomohisa Okuma, Akira Yamamoto, Masao Hamuro, Yukio Miki

**Affiliations:** Department of Radiology, Graduate School of medicine, Osaka City University, 1-4-3 Asahi-machi, Abeno-ku, Osaka, 545-8585 Japan; Depertment of Radiology, Osaka Saiseikai Nakatsu Hospital, 2-10-39 Sibata, Kita-ku, Osaka, 530-0012 Japan

**Keywords:** Radiofrequency ablation, Palliative treatment, Carcinomatous neuropathy

## Abstract

**Background:**

We present a case series of a palliative radiofrequency ablation (RFA) for the tumors that lead to the resolution of pain and motor function disorders. RFA is widely used on tumors in various organs and often reported in good outcome. There are some reports that RFA was performed as a palliative treatment but a few reports of RFA that performed for lung tumor as a palliative treatment. This case series includes two cases, palliative RFA for a sacrum and a lung tumor. The results of this case series presented that a palliative RFA is effective in improving the symptoms of patients.

**Case presentation:**

Case 1. A 64-year-old Japanese woman with a chordoma at her sacrum presented with pain in her left leg and claudication. Though operations, radiation therapy and GS-TAE (gelatin sponge–transarterial embolization, via the L5 lumbar artery) were performed, the size of the tumor leading pain and claudication increased. RFA was performed for the sacral tumor, and these symptoms resolved one year after the procedure.

Case 2. A 68-year-old Japanese man with a leiomyosarcoma at the apex of left lung presented with pain and motor function disorders of the left upper limb. Dissemination in the pleura was appeared after the operation for a leiomyosarcoma at the mediastinum. Though radiation therapy and a second operation were performed, the tumor at the apex of the left lung increased and pain and numbness of the left upper limb were appeared after the second operation. RFA was performed for the left lung tumor, and the symptoms resolved 3 months after RFA.

**Conclusion:**

RFA is effective as a palliative treatment and has a potential to salvage the patients from the symptoms of the tumors when conventional palliative treatments such as surgery, radiation therapy, and chemotherapy are difficult or contraindicated.

## Background

Radiofrequency ablation (RFA) is the treatment to malignant tumors by inserting an electrode into the tumor and causing the thermocoagulation necrosis of the tumor [1]. RFA is a minimally invasive therapy and has a low risk of major complications [2]. At present, RFA is widely used on tumors in various organs and results in good outcome [1, 3]. It has been reported that RFA is also effective for the palliative treatment [4]. A palliative RFA is performed when conventional palliative treatments such as surgery, radiation therapy, and chemotherapy are difficult or contraindicated. It has not previously been well established in the literature if a palliative RFA for the pulmonary tumor is effective. These cases consist of two patients, a sacrum tumor and pulmonary tumor with pain and neuropathy that received palliative RFA. In both cases, the symptoms resolved after the procedure.

## Case presentation

### Case 1

**Figure 1:**
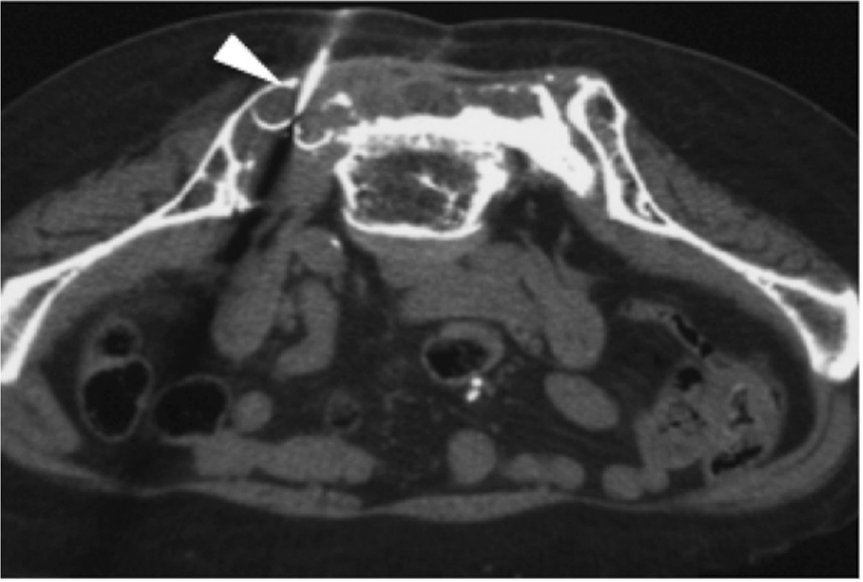
**Case 1:** **Non-** **contrast CT on the first session of tumor ablation.** A LeVeen electrode is located in the center of the tumor (arrowhead). This ablation was stopped before reaching “roll-off” because the patient complained of left leg pain and numbness.

**Figure 2:**
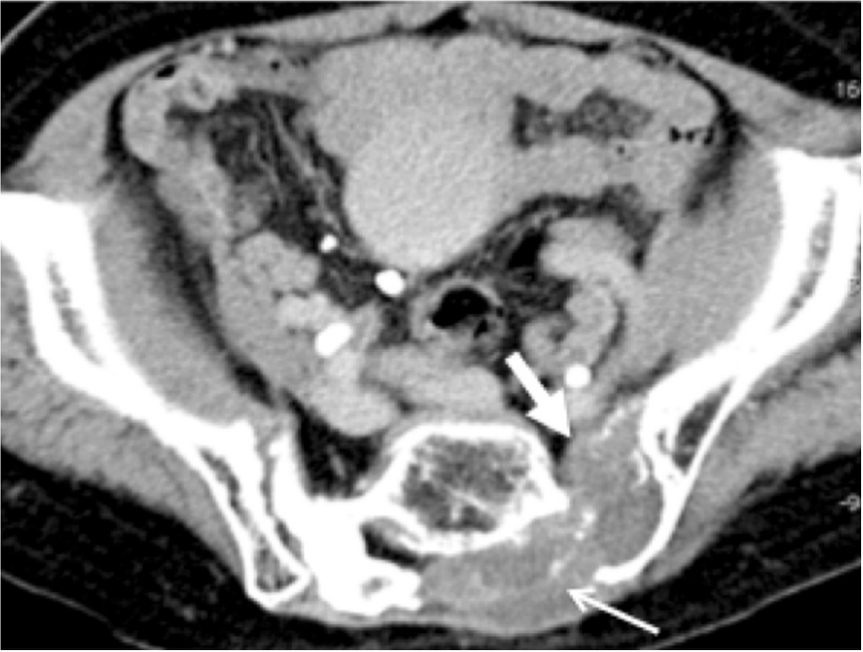
**Case 1:** **Contrast**-**enhanced CT about 1 year after RFA.** The sacral tumor did not increase (white arrow). However, the tumor progressed dorsally to the lateral of sacrum (white thin arrow).

A 64-year old Japanese woman underwent resection of a sacral chordoma. Recurrence of tumor appeared 7 years later, and thus reoperation was performed. Tumor recurrence was observed at 2 and again at 4 years after the reoperation. Therefore, radiation therapy (total dose: 51 Gy) and GS-TAE (gelatin sponge–transarterial embolization, via the L5 lumbar artery) were performed. However, the tumor increased in size and was accompanied by pain and claudication. Before RFA, she had been unable to sit due to complaints of pain and claudication in her left leg. Non-contrast computed tomography (CT) showed that the tumor was in the sacrum, and thus provided evidence that the symptoms were due to compression of the sciatic nerve.After informed consent was obtained RFA was performed. A 13-gauge needle was inserted in the cortex of the sacrum with CT guidance under local anesthesia. A LeVeen electrode was inserted coaxially in the tumor via a 13-gauge needle, and retractable hooks were opened (Figure 1). Ablation was performed four times, shifting the position of the electrode for every ablation. The starting output was 10 W, which was increased 10 W every 2 minutes. Ablation continued until the generator automatically stopped upon reaching the maximum resistance due to increased impedance (i.g. roll off). Ablation was started with the low output and if a sharp pain of her legs appeared, ablation was stopped in order to avoid the damage of nervous by heat. Total ablation time was 29 minutes and 39 seconds, and maximum output was 50 W. Ablation was interrupted every four cycles due to the pain in her left leg before “roll-off”.The pain and numbness in her left lower leg resolved immediately after the RFA procedure. The next day, the claudication had disappeared and it became possible for the patient to sit down. One year after RFA, the pain had not recurred and no increase in tumor size was revealed on CT (Figure 2).

### Case 2

**Figure 3:**
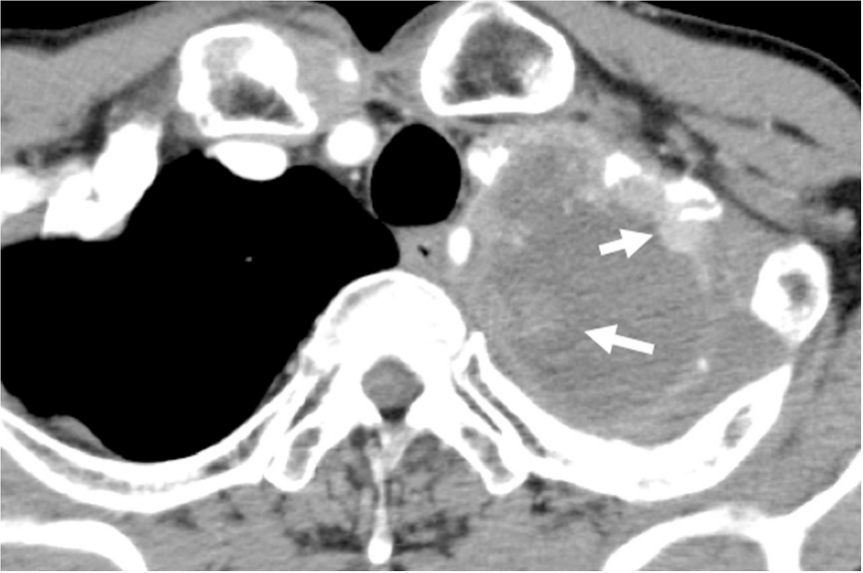
**Case 2: Contrast-** **enhanced CT pre**-**RFA.** A large tumor (over 10 cm in largest diameter) is observed in the apex of the left lung. This tumor contains a low-density area considered to be necrosis and enhanced solid lesion (white arrow).

**Figure 4:**
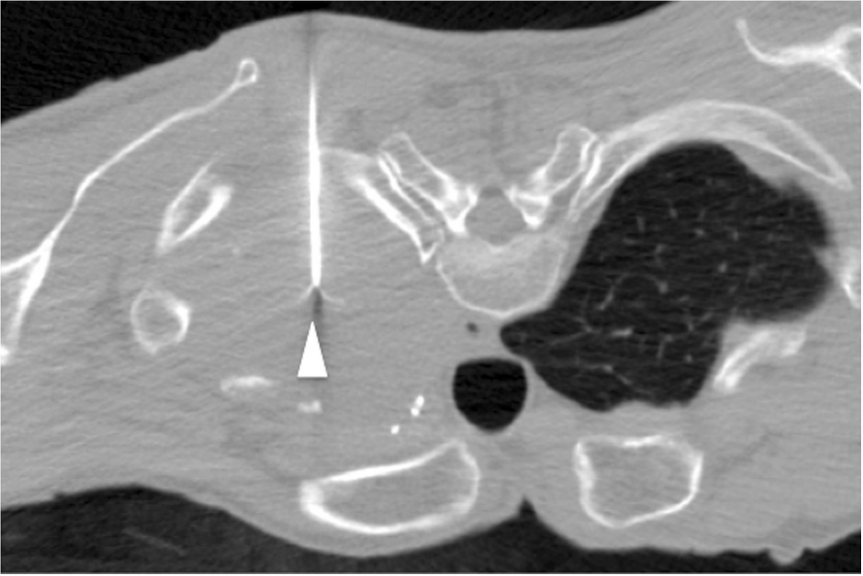
**Case 2: Non-** **contrast CT on the first ablation session.** A LeVeen electrode is located near the center of the tumor, and retractable hooks are fully opened (arrowhead). In this session, “roll-off” was not obtained even though ablation was continued for 20 minutes.

**Figure 5:**
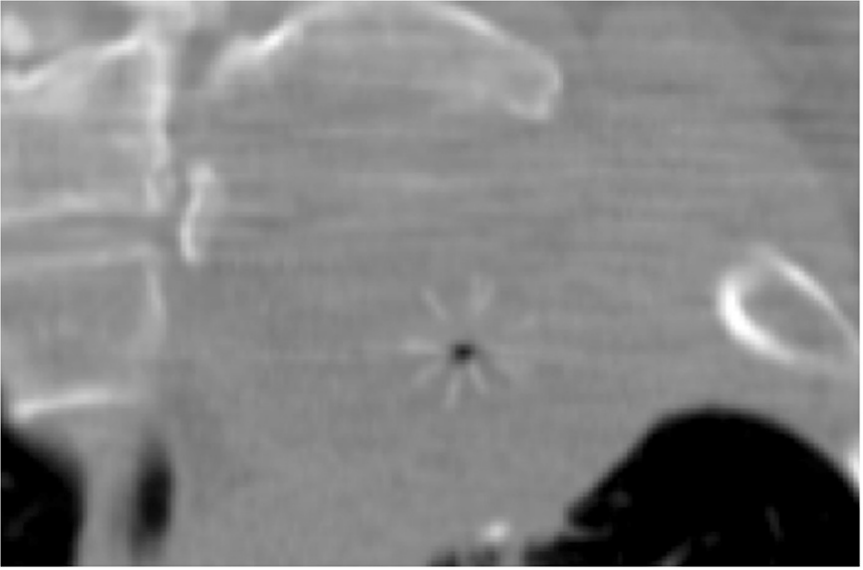
**Case 2: Non-** **contrast CT (coronal reformatted image) after the first ablation session.** The retractable hooks do not reach the chest wall.

**Figure 6:**
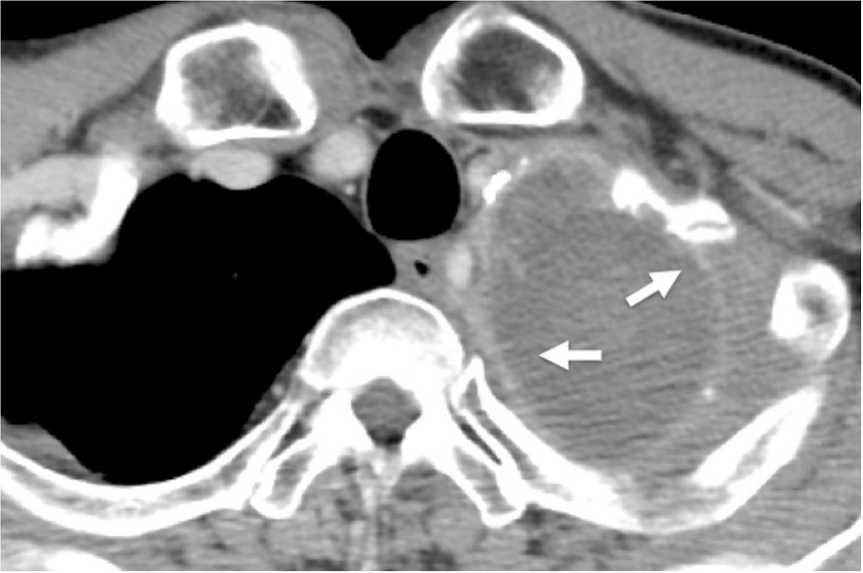
**Case 2: Contrast-** **enhanced CT 2 days after RFA.** Complications such as pneumothorax or hemorrhage are not observed. The intratumoral enhanced solid lesion is smaller compared to findings on pre-RFA CT (white arrow).

A 68-year-old Japanese man underwent resection of a leiomyosarcoma at the mediastinum. Dissemination in the pleura was observed, and thus radiation therapy (total dose: 40 Gy) and a second operation were performed. Pain and numbness of the left upper limb developed 10 months after the second operation and contrast enhanced CT scan was performed. Contrast enhanced CT scan showed a tumor at the apex of the left lung, accompanied by an enhanced solid lesion and a non-enhanced cystic lesion (Figure 3). The development of pain and numbness was observed at the lateral side of left antebrachium, digitus medius and anularis. The patients had the motor function disorder of left arm that he could not have or hold by left arm. RFA was chosen because it was thought that radiation and chemotherapy would not be effective and that surgery has a high risk of bleeding.RFA was performed with patient consent. A LeVeen electrode was inserted in the tumor with CT guidance and retractable hooks were opened (Figures 4, 5). The electrode was mostly located in the center of the tumor and it was confirmed that the electrode and plexus brachialis were fully separated on CT in order to avoid the damage of plexus brachialis by ablation. This procedure was performed under local anesthesia to monitor the appearance of sharp pain of left upper limb. The output was started at 20 W, and then increased by 10 W every 2 minutes. In each session, ablation continued until “roll off” and maximum ablation time was decided 20 minutes when “roll off” was not obtained. Ablation was interrupted if a sharp pain of left upper limb appeared. Ablation was performed three times. The output was started at 20 W, and then increased by 10 W every 2 minutes. Total ablation time was 57 minutes and 14 seconds, and maximum output was 80 W. “Roll-off” was obtained only in the third session of ablation.Contrast enhanced CT performed 2 days after RFA showed that the enhanced solid lesion in the tumor was smaller in size compared to pre-RFA CT (Figure 6). A pain had disappeared for about 3 months after RFA, accompanied by an improvement in motor function disorder, that the patient could extract the towel. However, 3 months after RFA, the pain of the left upper limb re-appeared due to growth of the tumor.

## Conclusion

Palliative RFA was performed to relieve symptoms induced by tumor in two cases, leading to the resolution of pain and motor function disorders in both. Therefore, RFA was found to be an effective palliative treatment.

At present, surgery for reduction of tumor volume, radiation therapy and chemotherapy have been performed as palliative treatments for relief of symptoms induced by tumors that cannot be radically resected [4]. However, these treatments have some limits. Surgical operation has a risk of bleeding and technical difficulties due to the size of tumors or adhesions. Radiation therapy is complicated by dose limitations in relation to re-irradiation, and it often takes several weeks before therapy is complete and relief of accompanying symptoms is achieved. Furthermore, radiation therapy has the risk of complications such as neuropathy and radiation pneumonitis [5].

Some reports have shown that ablation therapy is effective and safety palliative treatment for the relief of symptoms induced by tumor [4, 6]. Tumors can cause symptoms such as pain or motor function disorders in several ways. They can compress adjacent structures or nerves, increase intratumoral or interstitial pressure, or release cytotoxic substances [7]. RFA can provide pain relief through thermal coagulation necrosis and reduction of tumor volume. These processes subsequently reduce tumor compression of surrounding internal organs and nerves. Furthermore, coagulation necrosis leads to decreased production of cytotoxic substances released from the tumor [7].

Palliative RFA has some advantages compared to other palliative treatments. First, RFA is completed in 1 day and often provides rapid pain relief. Effects of RFA are observed immediately after the procedure and may rapidly improve quality of life. Second, RFA is minimally invasive and can be performed under local anesthesia. It has also been reported to have a low risk of major complications [2]. Furthermore, RFA can be performed repeatedly as long as the electrode puncturing procedure is technically possible. Repeat RFA has an opportunity to salvage tumors that progressed locally after the first RFA [8]. RFA may promote neuropathy since the temperature around the electrode rises and heat may spread in the adjacent structures (such as nerves). Therefore, physicians must monitor the ongoing possibility of neuropathy development during ablation. When neuropathy does develop, ablation of a tumor has to be aborted. In the cases of RFA tumor ablation presented in this report, the relief of symptoms were obtained though incomplete tumor ablation. Since palliative RFA is a medical treatment aimed at symptom relief, careful attention must be paid to the procedure.

Palliative RFA is an effective treatment for the relief of pain or the improvement of motor function disorders. Palliative RFA has a potential to salvage the patients from the symptoms of the tumors when conventional palliative treatments such as surgery, radiation therapy, and chemotherapy are difficult or contraindicated.

## Consent

Written informed consent was obtained from both patients for publication of this case report and accompanying images. A copy of the written consent is available for review by the Editor-in-Chief of this journal.
